# Enhanced Photoelectrochemical Performance of 2D Bi_2_O_3_/TiO_2_ Heterostructure Film by Bi_2_S_3_ Surface Modification and Broadband Photodetector Application

**DOI:** 10.3390/ma18153528

**Published:** 2025-07-28

**Authors:** Lai Liu, Huizhen Yao

**Affiliations:** 1 Key Laboratory of Instrumentation Science and Dynamic Measurement, School of Instrument and Electronics, North University of China, Taiyuan 030051, China; liulai@nuc.edu.cn; 2 Key Laboratory of Micro/Nano Devices and Systems, School of Semiconductor and Physics, North University of China, Taiyuan 030051, China

**Keywords:** Bi_2_S_3_/Bi_2_O_3_/TiO_2_, heterojunction, charge separation and transfer, PEC photodetector, photoelectrocatalysis

## Abstract

Photoelectrochemical devices have garnered extensive research attention in the field of smart and multifunctional photoelectronics, owing to their lightweight nature, eco-friendliness, and cost-effective manufacturing processes. In this work, Bi_2_S_3_/Bi_2_O_3_/TiO_2_ heterojunction film was successfully fabricated and functioned as the photoelectrode of photoelectrochemical devices. The designed Bi_2_S_3_/Bi_2_O_3_/TiO_2_ photoelectrochemical photodetector possesses a broad light detection spectrum ranging from 400 to 900 nm and impressive self-powered characteristics. At 0 V bias, the device exhibits an on/off current ratio of approximately 1.3 × 10^6^. It achieves a commendable detectivity of 5.7 × 10^13^ Jones as subjected to a 0.8 V bias potential, outperforming both bare TiO_2_ and Bi_2_O_3_/TiO_2_ photoelectrochemical devices. Moreover, the Bi_2_S_3_/Bi_2_O_3_/TiO_2_ photoelectrode film shows great promise in pollutant decomposition, achieving nearly 97.7% degradation efficiency within 60 min. The appropriate band energy alignment and the presence of an internal electric field at the interface of the Bi_2_S_3_/Bi_2_O_3_/TiO_2_ film serve as a potent driving force for the separation and transport of photogenerated carriers. These findings suggest that the Bi_2_S_3_/Bi_2_O_3_/TiO_2_ heterojunction film could be a viable candidate as a photoelectrode material for the development of high-performance photoelectrochemical optoelectronic devices.

## 1. Introduction

Photoelectrochemical (PEC) devices, possessing the remarkable ability of converting light signals into chemical fuels and electrical signals, hold great promise for applications such as sustainable fuel production [1], CO_2_ reduction [2,3], environmental pollutant degradation [4,5], advanced analytical systems [6], as well as emerging self-powered photodetectors [7,8,9]. The PEC devices can effectively integrate multiple functions, including degradation of pollutants and photodetection, into a single system, making them highly efficient and versatile in addressing various optical signal perceptions and environmental challenges. The operational mechanism of PEC devices encompasses the photoelectric effect [10], alongside the crucial separation and transport of photogenerated carriers. Moreover, the interfacial electrochemical reactions are notably also involved [11]. This synergistic interplay of physical and chemical processes affords more flexibility in adjusting the PEC performance. To achieve highly efficient PEC devices, it is significant to develop novel photoelectrode materials with good light harvesting ability and the desired spectral response range.

Bismuth oxide (Bi_2_O_3_), a pivotal p-type semiconductor, has emerged as an ideal photoelectrode material due to its suitable refractive index, high dielectric constant, air stability, low toxicity and simple preparation procedure [12,13,14,15,16]. Optoelectronic devices based on Bi_2_O_3_ semiconductor have been successfully developed. For example, Praveen et al. reported a flexible photodetector based on Bi_2_O_3_ nanofibers with a specific detectivity exceeding 10^9^ Jones [17]. Broadband Bi_2_O_3_ nanoparticles photodetector has also been realized by defect engineering, exhibiting high responsivity of 29.92 mA W^−1^ [18]. Nevertheless, rapid electron-hole pair recombination, restricted photon capture capacity and low transfer efficiency in Bi_2_O_3_ film still restrict the improvement of photoelectrical performance. Recently, massive efforts were carried out to overcome such issues, such as morphology modulation, doping with metal or non-metal ion, coupling with other semiconductors, including Bi_2_O_2_CO_3_, WO_3_, ZnO and TiO_2_ etc. [19,20,21,22,23,24,25,26,27,28]. Huang et al. successfully prepared Bi_2_O_3_/TiO_2_ heterojunction and achieved enhanced photoreactivity than pristine Bi_2_O_3_ [26]. It is verified that recombination of photoexcited electrons and holes was significantly reduced at the interface between Bi_2_O_3_ and TiO_2_. Sulfurization of the surface of metal oxide electrodes is an efficient technique for designing semiconductor heterojunctions or multi-component photoelectrodes [29]. Introducing bismuth-based sulfides in Bi_2_O_3_ film to construct Bi_2_S_3_/Bi_2_O_3_ composites can effectively improve its photoconductivity and reduce the lattice mismatch, which is beneficial to accelerate the transport of charge carrier [30]. Particularly, the staggered band energy alignment formed between Bi_2_O_3_ and Bi_2_S_3_ promotes photogenerated charge separation. Moreover, Bi_2_S_3_ semiconductors with narrow band gap (1.2–1.7 eV) have the ability to broaden the spectral response range of the photoelectrode [31]. Intriguingly, Bi_2_O_3_ can be easily transformed to Bi_2_S_3_ by direct and simple surface sulfurization reaction. For instance, Wang et al. prepared Bi_2_S_3_ nanopowders by sulfurization Bi_2_O_3_ microplates with excess thioacetamide as a sulfur source [32]. In PEC devices, there exists a contact interface between photoelectrode and electrolytes. Adjusting the morphology of Bi_2_O_3_ to two-dimensional (2D) nanoflakes with a substantial specific surface area can increase the solid-liquid interface and vastly facilitate charge transfer, thereby boosting PEC performance. The PEC device based on 2D Bi_2_O_3_ materials is expected to acquire high responsivity, fast response speed and high photoelectrocatalystic activity. However, the exploration on 2D Bi_2_O_3_-based PEC devices remains limited.

Herein, the efforts on the synthesis of Bi_2_S_3_/Bi_2_O_3_/TiO_2_ double heterojunctions were reported for the first time. The PEC activity of the effective photoelectrode was seriously investigated. The self-supported 2D nanosheets (NSs)/nanoflakes Bi_2_O_3_/TiO_2_ composites were firstly prepared by a two-step hydrothermal approach. Then, Bi_2_S_3_ layer was obtained through a fast, simple and mild anion exchange between Bi_2_O_3_ and sodium sulphide solution. The crystal structure, surface morphology, optical characteristics, microstructure, and element distribution of the as-prepared samples were systematically investigated. As-constructed Bi_2_S_3_/Bi_2_O_3_/TiO_2_ PEC device exhibits improved photodetection and good photoelectrocatalytic degradation performance. In addition, the photogenerated charge migration path in Bi_2_S_3_/Bi_2_O_3_/TiO_2_ composites and the work mechanism of the PEC device were discussed in detail. The findings demonstrate that the versatile heterojunction film can be a potential candidate for optical signal detection and environmental pollutant abatement.

## 2. Experimental Details

### 2.1. Materials

Fluoride-doped tin oxide (FTO, 2.2 mm, resistivity = 7 Ω/sq, transmittance ≥ 90%) glass substrates were purchased from Kaivo (Zhuhai, China). Acetone, isopropanol, ethanol, ethylene glycol (EG) and sodium sulfide (Na_2_S∙9H_2_O) were purchased from Sinopharm Chemical Reagent Co., Ltd. (Beijing, China). Tetrabutyl titanate (TBT), hydrochloric acid (HCl, 36~38%) and bismuth (III) nitrate pentahydrate (Bi(NO_3_)_3_∙5H_2_O) were purchased from Aladdin Co., Shanghai, China. Ammonium hexafluorotitanate (H_8_F_6_N_2_Ti) was purchased from Macklin incorporated company. All used reagents were analytical grade (AR) and without any additional purification. Aqueous solutions were prepared using ultra-pure deionized (DI) water with a resistivity of approximately 18 MΩ·cm.

### 2.2. Synthesis of TiO_2_ NSs Array Film

The TiO_2_ NSs array film grown on FTO substrate was fabricated by the hydrothermal method. Typically, FTO substrates with the dimensions of 1.0 × 3.0 cm^2^ were cleaned via sonication in acetone, isopropanol, ethanol and DI water for 20 min, respectively. The reaction precursor solution contained 30 mL of HCl/H_2_O (volume ratio, 1:1) and 0.8 mL of TBT. After stirring for 30 min, 0.5 g of H_8_F_6_N_2_Ti powder as a capping agent was added. A piece of cleaned FTO substrate was placed in a 100 mL Teflon-lined stainless-steel autoclave, followed by the addition of the homogeneous transparent solution. To synthesize TiO_2_ NSs film exposed to {001} facets with high reactive activity, the autoclave was transferred into a box oven and heated at 170 °C for 12 h. After cooling to atmospheric temperature naturally, the synthesized white film on FTO substrate was subjected to rinsing with DI water and then dried at 60 °C for 12 h in an oven. In the following step, the TiO_2_ NSs were rapidly placed into a preheated muffle furnace at 550 °C for 2 h to remove the fluorine species on the surface of TiO_2_ crystals.

### 2.3. Synthesis of Bi_2_S_3_/Bi_2_O_3_/TiO_2_ Dual Heterojunction Film

Firstly, the Bi_2_O_3_/TiO_2_ composites film was fabricated by hydrothermal method. The specific preparing process was as follows: 5 mM of Bi(NO_3_)_3_∙5H_2_O and 15 mL of EG were vigorously stirred to obtain a completely transparent solution. Subsequently, 30 mL of ethanol was dropped into the resultant solution. After stirring for 10 min, the mixed solution was poured into a 100 mL Teflon-lined stainless-steel autoclave. Then, FTO substrate with TiO_2_ NSs film was placed vertically in the autoclave. The hydrothermal fabrication procedure lasted for 6 h at the temperature of 160 °C. After the autoclave cooled to room temperature, the Bi_2_O_3_/TiO_2_ sample was rinsed with massive DI water and ethanol to remove other residues, and then dried at 60 °C for 12 h.

**Figure 1:**
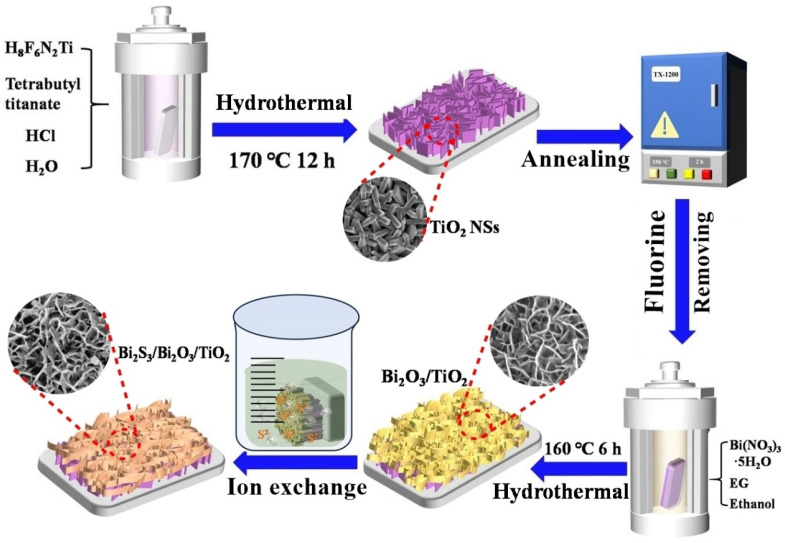
Schematic illustration for fabrication process of Bi_2_S_3_/Bi_2_O_3_/TiO_2_ heterojunction film by the hydrothermal and ion exchange method.

To grow Bi_2_S_3_ layer over the surface of Bi_2_O_3_/TiO_2_ film, in-situ anion exchange method was adopted. In a typical process, the prepared Bi_2_O_3_/TiO_2_ film was immersed in 0.1 M of Na_2_S∙9H_2_O solution for 5 min at room temperature. Then, the sample was taken out, thoroughly washed and dried under vacuum at 60 °C for 12 h. Figure 1 displays the schematic illustration of the synthetic process of the hierarchical heterostructure film comprising Bi_2_S_3_/Bi_2_O_3_/TiO_2_ on FTO substrate, achieved by a three-step method preparation process.

### 2.4. Characterization

Sample morphology was analyzed by field-emission scanning electron microscope (FESEM, MARA LMS, TESCAN). The crystal structure of as-prepared samples was performed on Rigaku D/max-2500 X-ray diffractometer using Cu Kα radiation (λ = 1.5418 Å) scanning from 20° to 80° with scanning rate of 2° min^−1^. The microstructure of the samples was analyzed through transmission electron microscopy (TEM, FEI, Tecnai-G2 F20) at 200 kV. Energy dispersive spectrometry (EDS) on TEM was performed for element mapping scanning. The surface chemical binding energy was determined by X-ray photoelectron spectroscopy (XPS, ESCLAB250Xi, Thermo Fisher Scientific, Waltham, MA, USA). The UV-visible (UV-vis) diffuse reflectance spectra of samples were performed on a Lambda 950 double-beam spectrophotometer to analyze the absorption property. The measurements were conducted with barium sulfate (Ba_2_SO_4_) as a reference material.

### 2.5. Photodetection Performance Measurements

Electrochemical workstation (CHI 660E) equipped with a three-electrode system in a quartz container was used to evaluate the PEC performance of the prepared photoelectrodes. The samples grown on FTO substrates were employed as the working photoelectrodes. Pt mesh played a role as a counter electrode, and the Ag/AgCl electrode was employed as a reference electrode, respectively. A 500 W xenon lamp was used as the simulated sunlight source. The photactive area of the working photoelectrode was maintained at 1 cm^2^ using a hard mask. The linear sweep voltammetry (LSV) was measured in 0.5 M KOH aqueous solution at a scan rate of 10 mV s^−1^. The transient photocurrent was performed by switching the light on and off. Electrochemical impedance spectroscopy (EIS) was measured at open circuit voltage with a frequency range from 0.01 to 100 kHz.

### 2.6. Photoelectrocatalytic Degradation of RhB

The photoelectrocatalytic activities of as-prepared pure TiO_2_ NSs, Bi_2_O_3_/TiO_2_ and Bi_2_S_3_/Bi_2_O_3_/TiO_2_ heterostructures were also assessed on a three-electrode system as mentioned above. The degradation process of Rhodamine B (RhB) was conducted under simulated sunlight irradiation from a 500 W xenon lamp equipped with a 420 nm cutoff filter. The characteristic absorption peak of RhB at 554 nm was used to determine the degradation ability of photoelectrodes. Typically, the work electrode was placed in 100 mL of supporting electrolyte with RhB (5 mg/L) and Na_2_SO_4_ (0.1 M) solution. Before light irradiation, the solution was magnetically stirred for 30 min in the dark to ensure an adsorption-desorption equilibrium between the RhB dye and photoelectrode. The degradation experiment was carried out at 0.8 V bias potential. 1 mL of mixed solution was taken out from the PEC reactor at 10 min intervals of illumination. After centrifugation at 8000 r/min for 5 min, the absorption behavior of the clear solution was measured by UV–vis spectrophotometer (UV-2600, Optical Instrument Factory, Shanghai, China).

## 3. Results and Discussion

**Figure 2:**
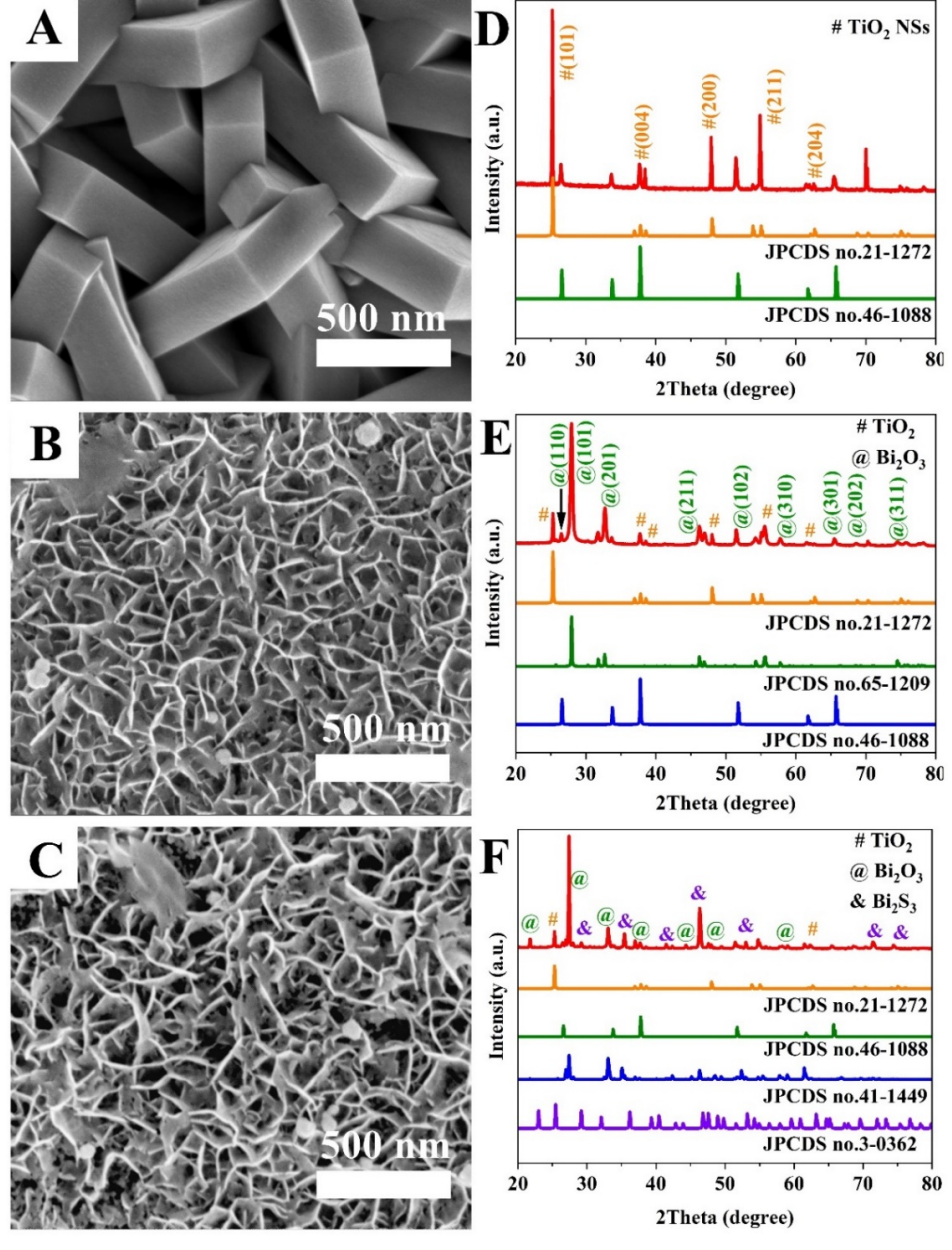
(**A**–**C**) SEM images of pristine TiO_2_ NSs, Bi_2_O_3_/TiO_2_, Bi_2_S_3_/Bi_2_O_3_/TiO_2_. (**D**–**F**) Corresponding XRD patterns.

We developed a two-step hydrothermal and ion-exchange method to fabricate Bi_2_S_3_/Bi_2_O_3_/TiO_2_ hierarchical film with dual heterostructure. The morphology of the obtained samples was investigated by FESEM technology, as shown in Figure 2A–C. The phase purity and crystal texture of the obtained samples were explored by XRD patterns (displayed in Figure 2D–F). Figure 2A shows the surface characteristics of 2D TiO_2_ NSs array film exposed to high-energy {001} facets with high reactive activity. It can be seen that the TiO_2_ nanocrystals cover uniformly and grow densely on the FTO substrate. The TiO_2_ NSs intercross with each other to form a network with sufficient internal surface area. The thickness of TiO_2_ NSs is close to 230 nm. Figure 2D is the corresponding XRD diffraction peaks of pristine TiO_2_ NSs. It can be found that the main characteristic peaks at 25.30°, 37.77°, 48.09°, 55.18° and 62.72° belong to the (101), (004), (200), (211) and (204) crystal planes of anatase phase TiO_2_ (JCPDS No. 21-1272). The sharp diffraction peaks verify its high crystallinity. Furthermore, other diffraction peaks are assigned to the FTO substrate (JCPDS No. 46-1088), indicating that the sample is highly purified. For the Bi_2_O_3_/TiO_2_ composite film, the SEM image in Figure 2B shows that a mass of Bi_2_O_3_ nanoflakes with the thickness of ~10 nm grows on the surface of TiO_2_ NSs. The XRD pattern of as-prepared Bi_2_O_3_/TiO_2_ film is shown in Figure 2E. All diffraction peaks marked in green perfectly correspond to the standard tetragonal phase Bi_2_O_3_ (JCPDS No. 65-1209) [33]. The Bi_2_O_3_ nanoflakes grown on TiO_2_ NSs have a preferential growth of (101) crystallographic. After experiencing the process of anion exchange, the objective Bi_2_S_3_/Bi_2_O_3_/TiO_2_ heterostructure film is obtained. The surface morphology of the sample is almost similar to that of Bi_2_O_3_/TiO_2_ film, as shown in Figure 2C. However, the space existing between the nanoflakes is distinctly enlarged, contributing to the corrosive effect on Bi_2_O_3_ nanoflakes in the anion-exchange process [34]. Meanwhile, it can be seen that the crystal phase structure of Bi_2_O_3_ nanoflakes is also transformed to monoclinic phase Bi_2_O_3_ (JCPDS No. 41-1449) after surface sulphuration, as shown in Figure 2F. Noticeably, it is also observed that new and obvious characteristic peaks (marked in purple) appear, which are matched to the orthorhombic phase Bi_2_S_3_ (JCPDS No. 3-0362). The main diffraction peaks located at 29.17° and 46.91° match well with the (320) and (501) planes of Bi_2_S_3_, respectively. The features strongly illustrate that the Bi_2_S_3_/Bi_2_O_3_/TiO_2_ heterojunction film has been successfully obtained.

**Figure 3:**
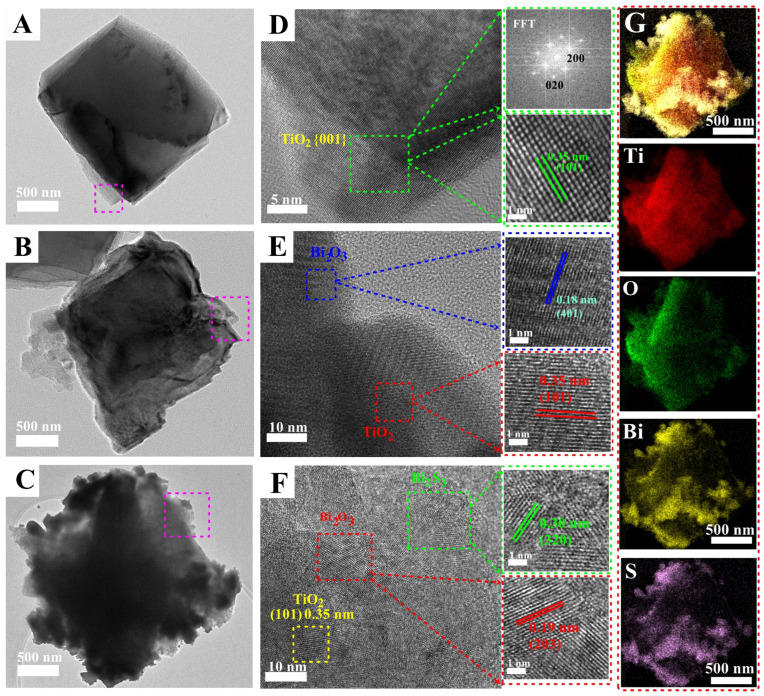
TEM images of (**A**) TiO_2_ NSs, (**B**) Bi_2_O_3_/TiO_2_ and (**C**) Bi_2_S_3_/Bi_2_O_3_/TiO_2_ composites film. (**D**), (**E**,**F**) Corresponding HRTEM images of as-prepared samples. Inset is FFT patterning of TiO_2_ NSs and corresponding enlarged HRTEM micrographs. (**G**) TEM-EDS mapping patterns of Bi_2_S_3_/Bi_2_O_3_/TiO_2_.

The detailed microstructures of pristine TiO_2_, Bi_2_O_3_/TiO_2_ and Bi_2_S_3_/Bi_2_O_3_/TiO_2_ films were further investigated by TEM and high-resolution TEM (HRTEM). TEM image in Figure 3A shows a tetragonal structure of a pristine TiO_2_ sample. After the growth of Bi_2_O_3_ and Bi_2_S_3_ layers, TiO_2_ NSs building units can also be easily identified, as shown in Figure 3B,C. The results suggest that the preparation process of Bi_2_O_3_ and Bi_2_S_3_ layers did not destroy the structure of TiO_2_ NSs. HRTEM image of pristine TiO_2_ is shown in Figure 3D. The measured lattice spacing of 0.35 nm (shown in the inset) is indexed to (101) atomic planes of anatase phase TiO_2_. The clear stripes verify its good crystalline. In addition, the fast Fourier transform (FFT) array pattern shown in the inset of Figure 3D suggests the monocrystalline characteristics of TiO_2_ NSs and can be indexed as [001] zone axis diffraction, which indicates that the square surfaces are high-reactive {001} facets. Figure 3B,E show the TEM and HRTEM images of Bi_2_O_3_/TiO_2_ sample, respectively. The surface of the sample becomes rough, as shown in Figure 3B. It is noticeable that besides the stripes from TiO_2_ (101) plane (marked in red in Figure 3E), the lattice fringes with d-spacing of 0.18 nm correspond to the (401) planes of Bi_2_O_3_, which provides the direct strong evidence of the formation of Bi_2_O_3_/TiO_2_ heterostructure. Figure 3C,F show TEM and HRTEM images of Bi_2_S_3_/Bi_2_O_3_/TiO_2_. The lattice spacings of 0.19 nm and 0.30 nm are indexed to the (203) plane of Bi_2_O_3_ layer and (320) plane of Bi_2_S_3_ layer, respectively. Figure 3G shows TEM-EDS mapping patterns of the Bi_2_S_3_/Bi_2_O_3_/TiO_2_ sample. The images clearly show the uniform distribution of Ti, O, Bi and S elements in the sample. The Ti element is mainly distributed in the core region, while O, Bi and S elements cover the entire region. The results confirm that Bi_2_S_3_ layer is interlaced in the Bi_2_O_3_ nanoflakes matrix and forms an interface heterostructure in Bi_2_S_3_/Bi_2_O_3_/TiO_2_ film.

**Figure 4:**
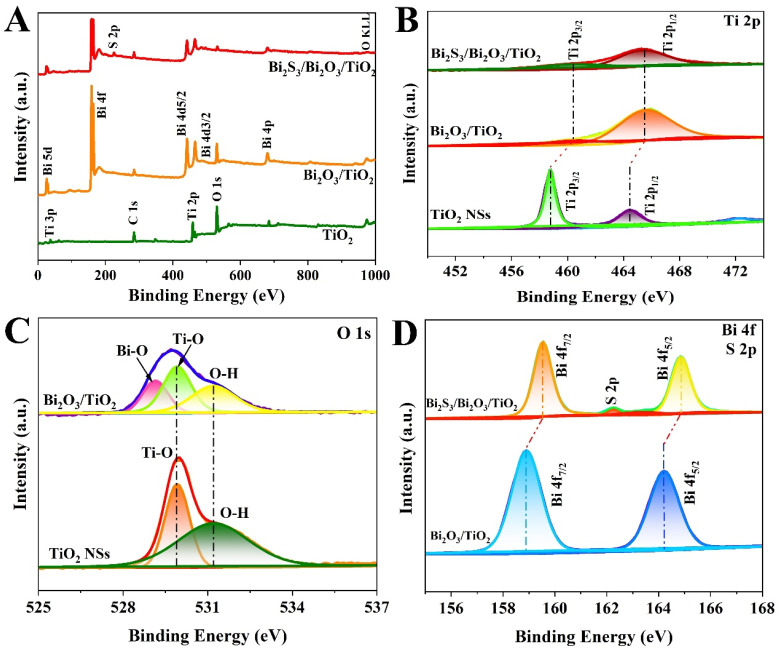
(**A**) Survey XPS spectra of TiO_2_ NSs, Bi_2_O_3_/TiO_2_ and Bi_2_S_3_/Bi_2_O_3_/TiO_2_ film. Corresponding high-resolution XPS spectra of (**B**) Ti 2p, (**C**) O 1s and (**D**) Bi 4f orbits.

The surface elemental configuration and chemical valence states of as-prepared TiO_2_, Bi_2_O_3_/TiO_2_ and Bi_2_S_3_/Bi_2_O_3_/TiO_2_ films were determined by XPS measurements. The binding energy of C 1s (at approximately 284.6 eV) was used to calibrate the XPS spectra from other elements. The C element comes from the carbon-based contaminant on the surface of samples [35]. Based on the survey spectra in Figure 4A, it can be observed that the Bi_2_S_3_/Bi_2_O_3_/TiO_2_ film is composed of Ti, O, Bi and S elements, coinciding with the analysis of EDS element mapping images displayed above. In the Ti 2p high-resolution scan spectrum of anatase TiO_2_ film (Figure 4B), the peaks at 458.78 and 464.65 eV are assigned to Ti^4+^ 2p_3/2_ and Ti^4+^ 2p_1/2_ energy levels, respectively. By contrast, the Ti 2p characteristic peaks in Bi_2_O_3_/TiO_2_ film shift to 460.48 and 465.30 eV. The shift is ascribed to the strong interaction between Bi_2_O_3_ nanoflakes and TiO_2_ NSs [36]. Furthermore, the characteristic peaks of Ti 2p of Bi_2_S_3_/Bi_2_O_3_/TiO_2_ are almost in line with that of Bi_2_O_3_/TiO_2_, suggesting that surface sulphuration by anion exchange method has little effect on the environment around Ti atom. O 1s spectra from pristine TiO_2_ and Bi_2_O_3_/TiO_2_ are shown in Figure 4C. Figure S1 (Supporting Information) shows that of Bi_2_S_3_/Bi_2_O_3_/TiO_2_ film. The O 1s spectrum of TiO_2_ film contains two peaks at 529.92 and 531.20 eV, corresponding to Ti–O bond and absorbed surface hydroxyl groups [37], respectively. It can be obviously observed that a new characteristic peak appears at approximately 529.15 eV in Bi_2_O_3_/TiO_2_ and Bi_2_S_3_/Bi_2_O_3_/TiO_2_ film, assigning to Bi–O bond. The results demonstrate the existence of Bi_2_O_3_ layer in composites film. The Bi 4f spectra of Bi_2_O_3_/TiO_2_ and Bi_2_S_3_/Bi_2_O_3_/TiO_2_ composites are shown in Figure 4D. There are two symmetrical peaks at 158.89 (Bi^3+^ 4f_7/2_) and 164.21 eV (Bi^3+^ 4f_5/2_) for Bi_2_O_3_/TiO_2_ film. While they move slightly to higher binding energy in Bi_2_S_3_/Bi_2_O_3_/TiO_2_ film, verifying interfacial charges transfer between Bi_2_S_3_ and Bi_2_O_3_ layer. The weak peak (at approximately 162.26 eV) between Bi 4f_7/2_ and Bi 4f_5/2_ of Bi_2_S_3_/Bi_2_O_3_/TiO_2_ is assigned to S 2p binding energy. In addition, the characteristic peak referred to S 2s (as shown in Figure S1, Supporting Information) can also be found in Bi_2_S_3_/Bi_2_O_3_/TiO_2_ film. The peak positioned at 225.4 eV is referred to Bi–S bond, functioning as a fast electron bridge for interface carrier transport. The peak located at 228.2 eV matches well with the S_8_ species [38]. The results further confirm the existence of Bi_2_S_3_ and Bi_2_O_3_ in the composite film.

**Figure 5:**
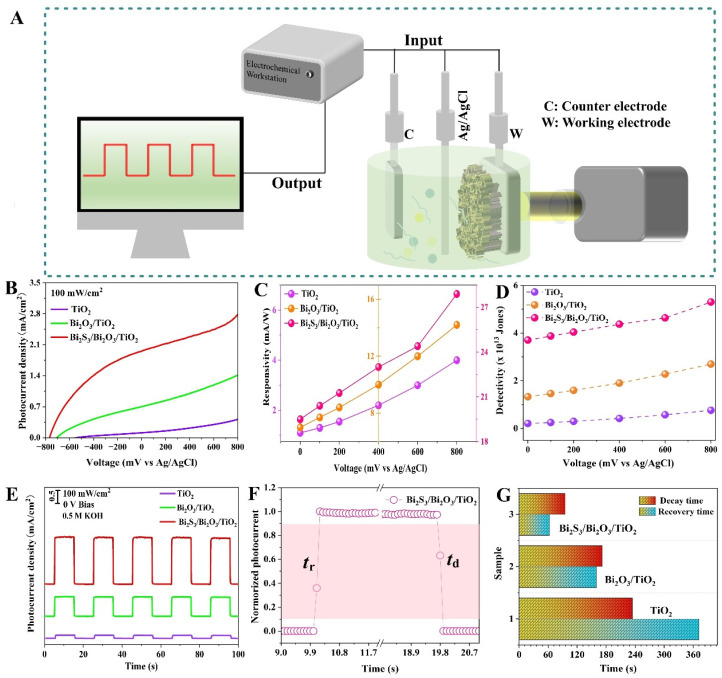
(**A**) Schematic illustration of the PEC photodetector and the three-electrode test system of the photodetection performance. Photodetection behaviors of TiO_2_, Bi_2_O_3_/TiO_2_ and Bi_2_S_3_/Bi_2_O_3_/TiO_2_ photoelectrodes in 0.5 M KOH solution under simulated sunlight illumination (**B**) LSV curves. (**C**,**D**) The corresponding calculated values of responsivity and detectivity at 100 mW cm^−2^. (**E**) Transient photocurrent at 0 V bias. (**F**) Rise and decay time of the device in one cycle. (**G**) Histogram of response time comparison for different devices.

To evaluate the potential photodetection application, a three-electrode system was constructed and the PEC performance of the devices based on TiO_2_, Bi_2_O_3_/TiO_2_ and Bi_2_S_3_/Bi_2_O_3_/TiO_2_ photoelectrode films was measured on an electrochemical workstation, as shown in Figure 5A. The LSV curves in 0.5 M KOH electrolyte under darkness are shown in Figure S2 (Supporting Information). The dark current (I_dark_) of Bi_2_S_3_/Bi_2_O_3_/TiO_2_ device is approximately 1.9 × 10^−6^ mA cm^−2^ at 0 V bias, an order of magnitude lower than those of TiO_2_ (6.8 × 10^−5^ mA cm^−2^) and Bi_2_O_3_/TiO_2_ (1.6 × 10^−5^ mA cm^−2^) devices. The phenomenon verifies a low leakage current in Bi_2_S_3_/Bi_2_O_3_/TiO_2_ device, which is attributed to the dual heterostructure formation and the reduced charge carrier recombination rate. Figure 5B shows the LSV curves of PEC devices under simulated sunlight illumination at 100 mW cm^−2^. All of the PEC devices display obvious photosensitivity to sunlight. The photocurrent density (I_light_) is almost stable within the test range, indicating the photoelectrodes do not experience electrochemical corrosion. In addition, the photocurrent of all three PEC devices shows obvious dependence on external bias potential. As shown in Figure S3 (Supporting Information), it can be found that the photocurrent of Bi_2_S_3_/Bi_2_O_3_/TiO_2_ device is always larger than those of the other devices at various bias potentials, which increases from 1.95 mA cm^−2^ at 0 V to 2.79 mA cm^−2^ at 0.8 V with an improvement of 43%. The sensitive characteristic to external bias potential gives flexibility to regulate photodetection performance in the actual usage scenario. The photoresponsivity (*R*) and photodetectivity (D*) are significant parameters to assess quantitatively the photoresponse performance of PEC photodetectors, which can be calculated using the following formulas [39,40]:

(1) $$R=\frac{I_{light}-I_{dark}}{P_{in}S}$$

(2) $$D^{*}=\frac{RS^{1/2}}{{\left(2eI_{dark}\right)}^{1/2}}$$

where *I*_light_ is the photocurrent density under illumination, *I*_dark_ is the current recorded in dark condition, *P*_in_ is the incident light power intensity, *S* is the effective area of the device and *e* is the elementary charge with a value of 1.60 × 10^−19^ C, respectively. Particularly, it can be found from Figure 5C,D that *R* and D* of the PEC devices display a gradual rising trend with an increasing bias potential. The phenomenon is attributing that separation and transport of photogenerated carriers can be efficiently promoted by the effect of external potential. As for the Bi_2_S_3_/Bi_2_O_3_/TiO_2_ device, the optimal *R* and D* reach up to 27.9 mA W^−1^ and 5.7 × 10^13^ Jones at 0.8 V, respectively. By contrast, the Bi_2_S_3_/Bi_2_O_3_/TiO_2_ device presents the best photoelectric performance, suggesting its more excellent ability of rapid charge carrier separation and transport.

Figure 5E shows the transient photocurrent density of various PEC devices at 0 V bias under periodic illumination at 10 s intervals. Impressively, the photocurrent density rapidly increases upon illumination and instantly decreases without light, indicating an obvious switching behavior. The photoresponse performance remains stable after several cycling tests, exhibiting their huge potential in self-powered device applications [41]. The light on/off ratio (*I*_light_/*I*_dark_) is another critical indicator to evaluate the performance of photodetectors. The calculated on/off ratio of PEC device based on Bi_2_S_3_/Bi_2_O_3_/TiO_2_ film is approximately 1.3 × 10^6^, higher that of Bi_2_O_3_/TiO_2_ (4.3 × 10^4^) and TiO_2_ (1.5 × 10^3^) devices, demonstrating Bi_2_S_3_/Bi_2_O_3_/TiO_2_ heterostructure composite film achieves superior photoelectrical performance. Moreover, the response and recovery characteristics were investigated to judge the response speed to external illumination, as shown in Figure 5F and Figure S4 (Supporting Information). The rise time (*t*_r_) is defined as the time interval for the photocurrent to increase from 10% to 90% of the saturated photocurrent, and the decay time (*t*_d_) refers to the time interval for the photocurrent to recover from 90% to 10% of the saturated photocurrent. The rise and decay times of Bi_2_S_3_/Bi_2_O_3_/TiO_2_ device are 63 ms and 95 ms, significantly superior to those of Bi_2_O_3_/TiO_2_ and TiO_2_ devices, as shown in Figure 5G. The results verify the high photoresponse speed of the Bi_2_S_3_/Bi_2_O_3_/TiO_2_ PEC device.

**Figure 6:**
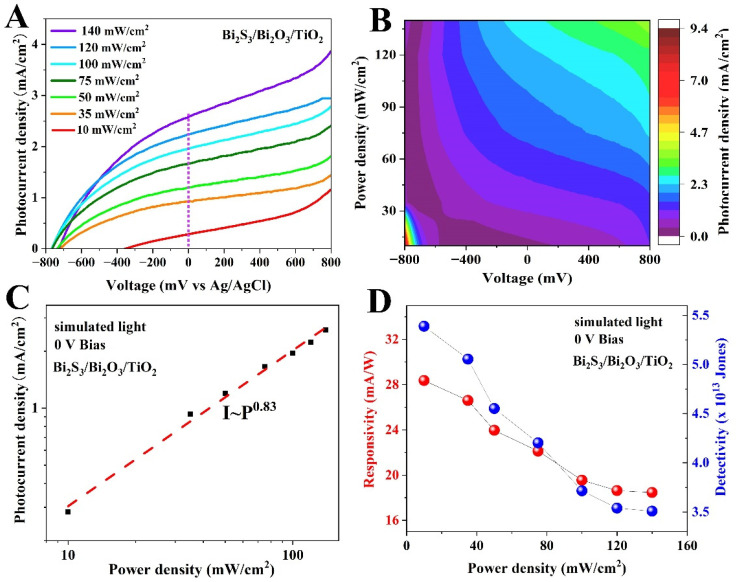
(**A**) LSV curves of Bi_2_S_3_/Bi_2_O_3_/TiO_2_ PEC devices under various light power densities. (**B**) Photocurrent density map as a function of both applied potential and light power density. (**C**) The photocurrent fitting curve is dependent on various light power intensities. (**D**) The corresponding calculated values of responsivity (red bullet) and detectivity (blue bullet).

**Figure 7:**
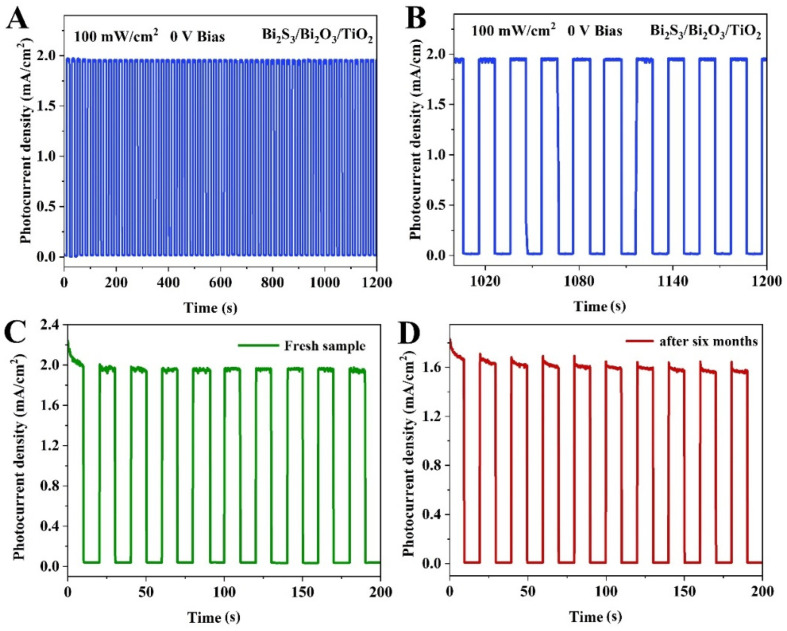
(**A**,**B**) Long-term stability test for 1200 s of the PEC device based on Bi_2_S_3_/Bi_2_O_3_/TiO_2_ composites film. (**C**) Transient photocurrent of a fresh sample at 0 V bias. (**D**) Corresponding transient photocurrent of sample storage for six months in air.

The photoresponse behaviors of Bi_2_S_3_/Bi_2_O_3_/TiO_2_ photodetector with various light power intensities were investigated. Figure 6A shows LSV curves of Bi_2_S_3_/Bi_2_O_3_/TiO_2_ PEC devices under different light power densities. It can be observed that the photocurrent gradually enhances as the bias potential increase with constant light intensity. Figure 6B displays a photocurrent density map as a function of both applied potential and light power density. It can be found that the photocurrent density also increases along with light power intensity, demonstrating a stable detecting ability. Specifically, the photocurrent at 0 V bias steadily increases from 0.28 to 2.58 mA cm^−2^ as the power intensity increases from 10 to 140 mW cm^−2^, resulting from more photogenerated charge in photoelectrode film at high light power intensity. As known, the dependence of the photocurrent (*I*) on the light power intensity (*P*) can be well fitted by the power law *I~P^θ^*. The exponent *θ* is determined by the photocurrent response to light intensity [42]. The *θ* value in the curve of Bi_2_S_3_/Bi_2_O_3_/TiO_2_ film shown in Figure 6C is determined as approximately 0.83. The linear dependence between the photocurrent density and the incident power indicates that there is an extremely low trap state density or an acceleration of detrapping dynamics with increasing carrier density. The calculated *R* and D* dependent on light power intensity is displayed in Figure 6D. With the increasing light power intensity, the values of *R* and D* present a downward trend and become saturated, which is attributed to the serious scattering or recombination rate of photogenerated carriers in higher incident power conditions [43,44]. When light power intensity is 100 mW cm^−2^, *R* and D* for the Bi_2_S_3_/Bi_2_O_3_/TiO_2_ film are approximately 19.6 mA W^−1^ and 3.7 × 10^13^ Jones at 0 V, respectively. The stability of the PEC photodetector is vital for the commercial practical application. Figure 7A shows the long-term stability of the Bi_2_S_3_/Bi_2_O_3_/TiO_2_ PEC device at the bias voltage of 0 V. Under the light intensity of 100 mW cm^−2^ for 1200 s, the photocurrent exhibits a negligible photocurrent density deficiency, demonstrating the excellent sustainability of the PEC device. Additionally, the storage stability of the device in air for six months is investigated, as shown in Figure 7C,D. After storage in air environment for six months, the photocurrent decreased to 1.56 mA cm^−2^, which may result from adsorbed humidity, oxygen or contamination from environment. The results suggest that the device has ultrahigh stability and huge application prospect. Moreover, a comparison of the performance metrics of Bi_2_S_3_/Bi_2_O_3_/TiO_2_ photodetectors has been summarized in Table 1. It can be found that the designed Bi_2_S_3_/Bi_2_O_3_/TiO_2_ PEC photodetector appears to have comparable photoresponsivity and response time. Noticeably, the detectivity of Bi_2_S_3_/Bi_2_O_3_/TiO_2_ PEC photodetector is superior to other reference devices.

**Figure 8:**
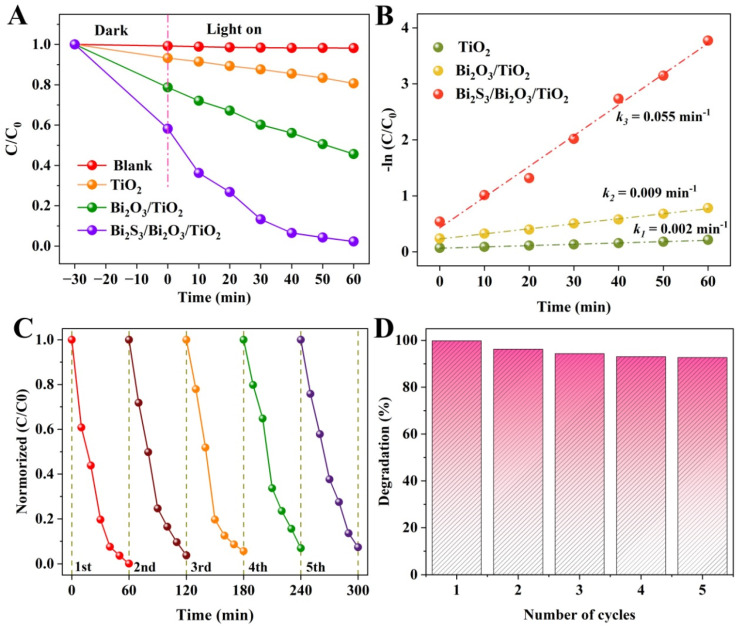
(**A**) RhB degradation of as-prepared samples under visible light irradiation. (**B**) Corresponding reaction kinetic curves. (**C**,**D**) Cyclic degradation curves and histogram for Bi_2_S_3_/Bi_2_O_3_/TiO_2_ film.

The photoelectrocatalytic activities of samples were evaluated by the degradation of RhB contaminant at 0.8 V bias. Figure 8A displays the degradation curves of the RhB contaminant with irradiation time over different photoelectrocatalysts. The blank test verifies that RhB dye is scarcely degraded by photolysis without photoelectocatalyst. The pristine TiO_2_ NSs film exhibits a weak photoelectrocatalytic activity and only degraded 20% of RhB in 60 min. While the RhB removal efficiency is boosted to 55% in the presence of Bi_2_O_3_/TiO_2_ photoelectrode, indicating that the formation of heterostructure is beneficial to improve photoelectrocatalytic performance. For Bi_2_S_3_/Bi_2_O_3_/TiO_2_ composites with dual heterojunctions, degrading efficiency rapidly reaches up to 75% after 20 min illumination. It is worth noting that the RhB dye is almost completely degraded within 60 min. The corresponding reaction kinetics of RhB degradation is shown in Figure 8B. All degradation experiments under illumination are found to follow the pseudo-first-order decay kinetics. The kinetics constants of TiO_2_, Bi_2_O_3_/TiO_2_ and Bi_2_S_3_/Bi_2_O_3_/TiO_2_ are 0.055, 0.009 and 0.002 min^−1^ for 0.8 V bias potential, respectively. The results indicate that the Bi_2_S_3_/Bi_2_O_3_/TiO_2_ composites achieve optimal photoelectrocatalytic degradation activity, suggesting the recombination of photogenerated electrons and holes is further suppressed. In addition, the self-supported Bi_2_S_3_/Bi_2_O_3_/TiO_2_ photoelectrocatalysts on the FTO substrate are recyclable and reusable. After five cycling runs (shown in Figure 8C,D), the RhB degradation efficiency still remained at 92%, demonstrating the good stability of the photoelectrode.

**Figure 9:**
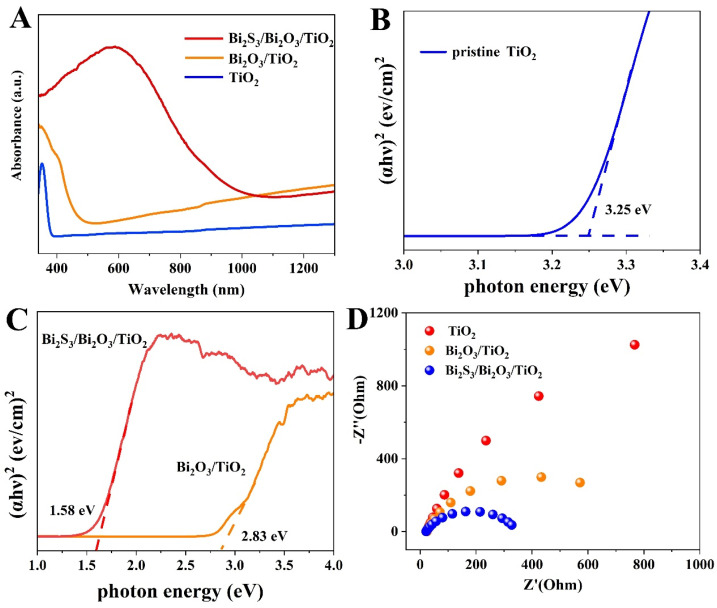
(**A**) UV-vis absorption spectra of the pristine TiO_2_, Bi_2_O_3_/TiO_2_ and Bi_2_S_3_/Bi_2_O_3_/TiO_2_. (**B**,**C**) Corresponding Tauc plots of samples. (**D**) Nyquist plots of electrochemical impedance spectra.

In our present work, the obtained Bi_2_S_3_/Bi_2_O_3_/TiO_2_ photoelectrode film reveals enhanced PEC performance compared with pure TiO_2_, and Bi_2_O_3_/TiO_2_ composites. The excellent PEC activity is attributed to the nature of Bi_2_S_3_ and Bi_2_O_3_ semiconductors. Figure 9A presents the UV-vis absorbance spectra of prepared samples. It can be found that pristine TiO_2_ film only exhibits an evident response to the ultraviolet light region due to its band gap of approximately 3.25 eV (shown in Figure 9B), similar to the previous report [26]. After being composited with Bi_2_O_3_ nanoflakes, the absorption range is broadened to approximately 500 nm. From the Tauc plot of Bi_2_O_3_/TiO_2_ displayed in Figure 9C, the band gap of Bi_2_O_3_ can be determined as approximately 2.83 eV, a similar value to that reported in the literature [26]. In contrast, the Bi_2_S_3_/Bi_2_O_3_/TiO_2_ composites film shows an obvious absorption between 400 and 900 nm, resulting from the coupled Bi_2_S_3_ layer on the surface with a band gap of approximately 1.58 V, close to the value in the previous report [31]. Moreover, the stronger absorption is found for the Bi_2_S_3_/Bi_2_O_3_/TiO_2_ film, which may be ascribed to the design of the hierarchical configuration in favor of light refraction and harvesting. As a result, the composite film can readily utilize low-energy photons and generate more charge carriers to improve the PEC performance. Electrochemical impedance spectroscopy (EIS) has been measured in order to investigate the transport behavior of charge carriers, as shown in Figure 9D. The radius is closely related to the charge transfer process at the photoelectrode-electrolyte interface. It is found that the Bi_2_S_3_/Bi_2_O_3_/TiO_2_ exhibits the smallest charge transfer resistance, benefitting from the migration of charge carriers. Therefore, the excellent charge-transfer capacity of Bi_2_S_3_/Bi_2_O_3_/TiO_2_ also contributes to the enhancement of photoresponse and photoelectrocatalysis performance.

**Figure 10:**
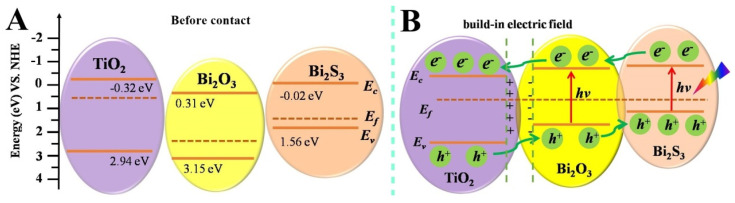
Schematic diagram for the formation of the dual heterojunctions and the possible charge separation and transport path.

To further explain the possible enhanced mechanism of PEC performance, the energy band structure in the Bi_2_S_3_/Bi_2_O_3_/TiO_2_ composite film is investigated in detail. The energy band alignment between Bi_2_S_3_, Bi_2_O_3_ and TiO_2_ plays a significant role in separating the photogenerated electron and hole pair. The specific conduction band (CB) and valence band (VB) edge potentials of the three semiconductors can be predicted by the following empirical equations [50]:

(3) $$E_{\mathrm{CB}}\;=\;X\;-\;\;E_{0}\;-\;0.5E_{g}$$

(4) $$E_{\mathrm{VB}}=X\;-\;E_{0}+0.5E_{g}$$

where X is the absolute electronegativity of the semiconductor, *E*_0_ is the energy of free electrons on the hydrogen scale (~4.5 eV), and *E*_g_ is the band gap of the semiconductor. The X values for TiO_2_, Bi_2_O_3_ and Bi_2_S_3_ are 5.81, 6.23 and 5.27 eV, respectively. Based on the formulas mentioned above, the calculated values of *E*_CB_, *E*_VB_ for TiO_2_, Bi_2_O_3_ and Bi_2_S_3_ semiconductors are listed in Table S1 (Supporting Information). On the basis of the relevant values, the energy band structure of the TiO_2_, Bi_2_O_3_ and Bi_2_S_3_ before contact is shown in Figure 10A. Commonly, TiO_2_ is considered as an intrinsic n-type semiconductor [51], its Fermi energy level approaches to the conduction band. While Bi_2_O_3_ and Bi_2_S_3_ are considered as p-type semiconductors, so the Fermi level is close to the valence band. After the semiconductors are closely connected together, the Fermi level in TiO_2_ NSs shift downward and those in Bi_2_O_3_ and Bi_2_S_3_ rise, until they reach a thermal equilibrium Fermi level [26]. Meanwhile, their valence and conduction bands undergo realignment and form a built-in electric field. The descended conduction band and ascended valence band indicate that type-II type heterojunction is established in the Bi_2_S_3_/Bi_2_O_3_/TiO_2_ composite film, as shown in Figure 10B. Under illumination, Bi_2_O_3_ and Bi_2_S_3_ with a relatively narrow band gap are excited to produce charge carriers. The photogenerated electrons on the conduction band of the p-type Bi_2_S_3_ and Bi_2_O_3_ transfer to that of n-type TiO_2_, whereas the in the valence band of TiO_2_ are transferred to the valence band of Bi_2_O_3_ and Bi_2_S_3_ under the effect of the built-in electric field. As discussed above, the p-n heterojunction inhibits the recombination of photogenerated charge carriers, which contributes to more photogenerated carriers participating in PEC reactions for photodetection and photoelectrocatalysis. Specifically, holes accumulated on the surface of Bi_2_S_3_ layer oxidize OH^−^ anions in the electrolytes to form OH*, as shown in Figure S5 (Supporting Information). Electrons accumulated on the surface of TiO_2_ swiftly move to the FTO conductive substrate and transport to the counter electrode (Pt) through the external circuit. At the solid-liquid interface, the photogenerated electrons reduce the radical OH* to form OH^−^ anions. The movement of photogenerated electrons generates the electric signal, which can realize the function of photodetection. In the photoelectrocatalystic degradation process, electrons are captured by oxygen adsorbed on the surface of Pt electrode to generate O_2_^−^. Meanwhile, the holes gather at the photoelectrode/electrolyte interface and can directly oxidize the RhB dye or cause indirect oxidation through the generation of oxidizing species such as the hydroxyl radical. According to the above analysis, the enhanced PEC performance of Bi_2_S_3_/Bi_2_O_3_/TiO_2_ film is mainly due to the formation of dual heterostructures and the broadband light response.

## 4. Conclusions

In summary, we have demonstrated a two-step hydrothermal method to synthesize hierarchical Bi_2_O_3_/TiO_2_ heterojunction film. After in-situ surface sulphuration, the Bi_2_S_3_ layer was successfully incorporated onto the surface of Bi_2_O_3_/TiO_2_ composites_,_ forming dual heterojunctions that promote both charge migration and light harvesting with the reduced recombination of charge carriers. The composite film exhibits excellent PEC activity with high stability under illumination, which is much higher than that of pure TiO_2_ and Bi_2_O_3_/TiO_2_ composites. The light on/off current ratio of Bi_2_S_3_/Bi_2_O_3_/TiO_2_ device approximately reaches to 1.3 × 10^6^ at 0 V bias. Furthermore, the photoelectrode film demonstrated approximately 97.7% degradation after 60 min of PEC reaction. This comprehensive investigation enhances our understanding of developing high-performance optoelectronic devices and paves the way for advancements in multifunctional PEC devices.

## Figures and Tables

**Table 1:** Performance comparison with other bismuth-based PEC photodetectors.

Materials	Wavelength (nm)	Electrolyte	Response (ms)	R (mA W^−1^)	D* (10^12^ Jones)	Refs.
Bi_2_S_3_/Bi_2_O_3_/TiO_2_	Sunlight	0.5 M KOH	63/95	19.5	37	This work
BiVO_4_/MXene	Sunlight	1 M Na_2_SO_3_ + 0.5 M phosphate	8/14	40.95	-	[8]
Bi_2_O_2_S	365	1 M KOH	10/45	13	2.34 × 10^−2^	[45]
2D Bi_2_S_3_	400	0.1 M KOH	100/100	0.7	3.75 × 10^−4^	[46]
Bi QDs	350	1 M KOH	100/200	0.295	9.09 × 10^−4^	[47]
Bi_2_O_3_/CuBi_2_O_4_	380	0.3 M K_2_SO_4_ + 0.2 M phosphate	0.18/0.19	75	-	[48]
SnS_2_/BiVO_4_	500	1 M Na_2_SO_3_ + 0.5 M phosphate	6/21	10.43	-	[49]

## Data Availability

The original contributions presented in this study are included in the article/Supplementary Material. Further inquiries can be directed to the corresponding author.

## Supplementary Materials

The following supporting information can be downloaded at: https://www.mdpi.com/article/10.3390/ma18153528/s1, Figure S1: High-resolution XPS spectra of O 1s and S 2s in Bi_2_S_3_/Bi_2_O_3_/TiO_2_ film. Figure S2: LSV curves under dark. Figure S3: Histogram of photocurrent density at various bias potential for different PEC devices. Figure S4: Transient photocurrent at 0 V bias of (A) pristine TiO_2_ and Bi_2_O_3_/TiO_2_ film. Table S1: Calculated E_CB_ and E_VB_ of TiO_2_, Bi_2_O_3_ and Bi_2_S_3_. Figure S5: Schematic illustration of PEC photodetector based on Bi_2_S_3_/Bi_2_O_3_/TiO_2_ composites film.

## References

[1] Lianos P. (2017). Review of recent trends in photoelectrocatalytic conversion of solar energy to electricity and hydrogen. Appl. Catal. B Environ..

[2] Xu Y., Wang F., Lei S., Wei Y., Zhao D., Gao Y., Ma X., Li S., Chang S., Wang M. (2023). In situ grown two-dimensional TiO_2_/Ti_3_CN MXene heterojunction rich in Ti^3+^ species for highly efficient photoelectrocatalytic CO_2_ reduction. Chem. Eng. J..

[3] Sorokina L., Savitskiy A., Shtyka O., Maniecki T., Szynkowska-Jozwik M., Trifonov A., Pershina E., Mikhaylov I., Dubkov S., Gromov D. (2022). Formation of Cu-Rh alloy nanoislands on TiO_2_ for photoreduction of carbon dioxide. J. Alloys Compd..

[4] Wang W., Liu X., Jing J., Mu J., Wang R., Du C., Su Y. (2023). Photoelectrocatalytic peroxymonosulfate activation over CoFe_2_O_4_-BiVO_4_ photoanode for environmental purification: Unveiling of multi-active sites, interfacial engineering and degradation pathways. J. Colloid Interface Sci..

[5] Xiong Y., Ma S., Hong X., Long J., Wang G. (2023). Photoelectrocatalytic Processes of TiO_2_ Film: The Dominating Factors for the Degradation of Methyl Orange and the Understanding of Mechanism. Molecules.

[6] García-Ramírez P., Pineda-Arellano C.A., Millán-Ocampo D.E., Álvarez-Gallegos A., Sirés I., Silva-Martínez S. (2024). Photoelectrocatalytic chemical oxygen demand analysis using a TiO_2_ nanotube array photoanode. Electrochim. Acta.

[7] Liu X., Wang D., Shao P., Sun H., Fang S., Kang Y., Liang K., Jia H., Luo Y., Xue J. (2022). Achieving record high external quantum efficiency >86.7% in solar-blind photoelectrochemical photodetection. Adv. Funct. Mater..

[8] Zhou S., Jiang C., Han J., Mu Y., Gong J.R., Zhang J. (2025). High-Performance self-powered PEC photodetectors based on 2D BiVO_4_/MXene Schottky Junction. Adv. Funct. Mater..

[9] Ma Y., Huang Y., Huang J., Xu Z., Yang Y., Xie C., Zhang B., Ao G., Fu Z., Li A. (2024). Optimizing Photoelectrochemical UV Imaging Photodetection: Construction of Anatase/Rutile Heterophase Homojunctions and Oxygen Vacancies Engineering in MOF-Derived TiO_2_. Molecules.

[10] Shu J., Tang D. (2020). Recent advances in photoelectrochemical sensing: From engineered photoactive materials to sensing devices and detection modes. Anal. Chem..

[11] Leng W.H., Zhang Z., Zhang J.Q., Cao C.N. (2005). Investigation of the kinetics of a TiO_2_ photoelectrocatalytic reaction involving charge transfer and recombination through surface states by electrochemical impedance spectroscopy. J. Phys. Chem. B.

[12] Sun C., Wu L., Hu J., Hussain S.A., Yang J., Jiao F. (2023). A novel dual S-scheme heterojunction photocatalyst β-Bi_2_O_3_/NiAl-LDH/α-Bi_2_O_3_ induced by phase-transformed bismuth oxide for efficient degradation of antibiotics in full-spectrum: Degradation pathway, DFT calculation and mechanism insight. Chem. Eng. J..

[13] Shanmugapriya V., Arunpandiyan S., Hariharan G., Bharathi S., Selvakumar B., Arivarasan A. (2022). Enhanced electrochemical performance of mixed metal oxide (Bi_2_O_3_/ZnO) loaded multiwalled carbon nanotube for high-performance asymmetric supercapacitors. J. Energy Storage.

[14] Feng X., Zou H., Zheng R., Wei W., Wang R., Zou W., Lim G., Hong J., Duan L., Chen H. (2022). Bi_2_O_3_/BiO_2_ nanoheterojunction for highly efficient electrocatalytic CO_2_ reduction to formate. Nano Lett..

[15] Zhang J., Xiong Z., Wang Z., Sun J. (2024). Study on the Preparation and PEC-Type Photodetection Performance of β-Bi_2_O_3_ Thin Films. Materials.

[16] Liu X., Sun Z., Sun Y., Lin H., Chen Z., Chen X., Niu L., Zhang Q., Li H. (2023). Fast and Long-Lasting Potassium-Ion Storage Enabled by Rationally Engineering Strain-Relaxation Bi/Bi_2_O_3_ Nanodots Embedded in Carbon Sheets. Adv. Funct. Mater..

[17] Praveen S., Veeralingam S., Badhulika S. (2021). A Flexible Self-Powered UV Photodetector and Optical UV Filter Based on β-Bi_2_O_3_/SnO_2_ Quantum Dots Schottky Heterojunction. Adv. Mater. Interfaces.

[18] Yang S., Jiao S., Nie Y., Lu H., Liu S., Zhao Y., Gao S., Wang D., Wang J., Li Y. (2022). A self-powered high performance UV-Vis-NIR broadband photodetector based on β-Bi_2_O_3_ nanoparticles through defect engineering. J. Mater. Chem. C.

[19] Guo P., Yin F., Zhang J., Chen B., Ni Z., Shi L., Han M., Wu Z., Li G. (2024). Crystal-Phase and Surface-Structure Engineering of Bi_2_O_3_ for Enhanced Electrochemical N_2_ Fixation to NH_3_. ACS Appl. Mater. Interfaces.

[20] Liu Y., Chu C., Li Y., Deng P., Liu Y., Wu R., Liu X., Zheng Y., Zhang W., Wu J. (2022). Enhanced supercapacitor performance of Bi_2_O_3_ by Mn doping. J. Alloys Compd..

[21] Hu R., Xiao X., Tu S., Zuo X., Nan J. (2015). Synthesis of flower-like heterostructured β-Bi_2_O_3_/Bi_2_O_2_CO_3_ microspheres using Bi_2_O_2_CO_3_ self-sacrifice precursor and its visible-light-induced photocatalytic degradation of *o*-phenylphenol. Appl. Catal. B Environ..

[22] Khan I., Abdalla A., Qurashi A. (2017). Synthesis of hierarchical WO_3_ and Bi_2_O_3_/WO_3_ nanocomposite for solar-driven water splitting applications. Int. J. Hydrogen Energy.

[23] Yasin M., Saeed M., Muneer M., Usman M., Haq A.U., Sadia M., Altaf M. (2022). Development of Bi_2_O_3_-ZnO heterostructure for enhanced photodegradation of rhodamine B and reactive yellow dyes. Surf. Interfaces.

[24] Wang P., Wang S.-Z., Kang Y.-R., Sun Z.-S., Wang X.-D., Meng Y., Hong M.-H., Xie W.-F. (2021). Cauliflower-shaped Bi_2_O_3_–ZnO heterojunction with superior sensing performance towards ethanol. J. Alloys Compd..

[25] Balachandran S., Swaminathan M. (2012). Facile fabrication of heterostructured Bi_2_O_3_–ZnO photocatalyst and its enhanced Photocatalytic Activity. J. Phys. Chem. C.

[26] Huang Y., Wei Y., Wang J., Luo D., Fan L., Wu J. (2017). Controllable fabrication of Bi_2_O_3_/TiO_2_ heterojunction with excellent visible-light responsive photocatalytic performance. Appl. Surf. Sci..

[27] Sood S., Mehta S.K., Sinha A.S.K., Kansal S.K. (2016). Bi_2_O_3_/TiO_2_ heterostructures: Synthesis, characterization and their application in solar light mediated photocatalyzed degradation of an antibiotic, ofloxacin. Chem. Eng. J..

[28] Wang C., Tan C., Lv W., Zhu G., Wei Z., Zhang K.H.L., He W. (2018). Coherent Bi_2_O_3_/TiO_2_ heterojunction material through oriented growth as an efficient photocatalyst for methyl orange degradation. Mater. Today Chem..

[29] Taghinejad H., Taghinejad M., Abdollahramezani S., Li Q., Woods E.V., Tian M., Eftekhar A.A., Lyu Y., Zhang X., Ajayan P.M. (2025). Ion-assisted nanoscale material engineering in atomic layers. Nano Lett..

[30] Huang Y., Fan W., Long B., Li H., Zhao F., Liu Z., Tong Y., Ji H. (2016). Visible light Bi_2_S_3_/Bi_2_O_3_/Bi_2_O_2_CO_3_ photocatalyst for effective degradation of organic pollutions. Appl. Catal. B: Environ..

[31] Bhoi Y.P., Mishra B.G. (2017). Single step combustion synthesis, characterization and photocatalytic application of α-Fe_2_O_3_-Bi_2_S_3_ heterojunctions for efficient and selective reduction of structurally diverse nitroarenes. Chem. Eng. J..

[32] Ke J., Liu J., Sun H., Zhang H., Duan X., Liang P., Li X., Tade M.O., Liu S., Wang S. (2017). Facile assembly of Bi_2_O_3_/Bi_2_S_3_/MoS_2_ n-p heterojunction with layered n-Bi_2_O_3_ and p-MoS_2_ for enhanced photocatalytic water oxidation and pollutant degradation. Appl. Catal. B Environ..

[33] Shinde N.M., Xia Q.X., Yun J.M., Shinde P.V., Shaikh S.M., Sahoo R.K., Mathur S., Mane R.S., Kim K.H. (2019). Ultra-rapid chemical synthesis of mesoporous Bi_2_O_3_ micro-sponge-balls for supercapattery applications. Electrochim. Acta.

[34] Manjunatha C., Rastogi C.K., Rao B.M., Kumar S.G., Varun S., Raitani K., Maurya G., Karthik B., Swathi C., Sadrzadeh M. (2024). Advances in hierarchical inorganic nanostructures for efficient solar energy harvesting systems. ChemSusChem.

[35] Mangolini F., McClimon J.B., Rose F., Carpick R.W. (2014). Accounting for nanometer-thick adventitious carbon contamination in X-ray absorption spectra of carbon-based materials. Anal. Chem..

[36] Guo X., Liu S., Wang W., Li C., Yang Y., Tian Q., Liu Y. (2021). Plasmon-induced ultrafast charge transfer in single-particulate Cu_1.94_S–ZnS nanoheterostructures. Nanoscale Adv..

[37] Potlog T., Dumitriu P., Dobromir M., LuCa D. (2014). XRD and XPS Analysis of TiO_2_ Thin Films Annealed in Different Environments. J. Mater. Sci. Eng..

[38] Zingg D.S., Hercules D.M. (1978). Electron spectroscopy for chemical analysis studies of lead sulfide oxidation. J. Phys. Chem..

[39] Ma D., Zhao J., Wang R., Xing C., Li Z., Huang W., Jiang X., Guo Z., Luo Z., Li Y. (2019). Ultrathin GeSe Nanosheets: From Systematic Synthesis to Studies of Carrier Dynamics and Applications for a High-Performance UV–Vis Photodetector. ACS Appl. Mater. Interfaces.

[40] Vashishtha P., Prajapat P., Sharma A., Singh P., Walia S., Gupta G. (2023). Self-driven UVC–NIR broadband photodetector with high-temperature reliability based on a coco palm-like MoS_2_/GaN heterostructure. ACS Appl. Electron. Mater..

[41] Vashishtha P., Abidi H.I., Giridhar P.S., Verma K.A., Prajapat P., Bhoriya A., Murdoch B.J., Tollerud J.O., Walia S. (2024). CVD-grown monolayer MoS_2_ and GaN thin film heterostructure for a self-powered and bidirectional photodetector with an extended active spectrum. ACS Appl. Mater. Interfaces.

[42] Kim H.-S., Kumar M.D., Kim J., Lim D. (2018). Vertical growth of MoS_2_ layers by sputtering method for efficient photoelectric application. Sens. Actuators A Phys..

[43] Vashishtha P., Prajapat P., Kumar K., Kumar M., Walia S., Gupta G. (2023). Multiband spectral response inspired by ultra-high responsive thermally stable and self-powered Sb_2_Se_3_/GaN heterojunction based photodetector. Surf. Interfaces.

[44] Vashishtha P., Tanwar R., Gautam S., Goswami L., Kushwaha S.S., Gupta G. (2024). Wavelength-modulated polarity switch self-powered Bi_2_Se_3_/GaN heterostructure photodetector. Mater. Sci. Semicond. Process..

[45] Yang X., Qu L., Gao F., Hu Y., Yu H., Wang Y., Cui M., Zhang Y., Fu Z., Huang Y. (2022). High-Performance broadband photoelectrochemical photodetectors Based on Ultrathin Bi_2_O_2_S Nanosheets. ACS Appl. Mater. Interfaces.

[46] Huang W., Xing C., Wang Y., Li Z., Wu L., Ma D., Dai X., Xiang Y., Li J., Fan D. (2018). Facile fabrication and characterization of two-dimensional bismuth(iii) sulfide nanosheets for high-performance photodetector applications under ambient conditions. Nanoscale.

[47] Xing C., Huang W., Xie Z., Zhao J., Ma D., Fan T., Liang W., Ge Y., Dong B., Li J. (2018). Ultrasmall Bismuth Quantum Dots: Facile liquid-Phase exfoliation, characterization, and application in high-performance UV–Vis photodetector. ACS Photonics.

[48] Dong B., Zhang X., Cheng H., Jiang X., Wang F. (2023). Ultrathin CuBi_2_O_4_ on a bipolar Bi_2_O_3_ nano-scaffold: A self-powered broadband photoelectrochemical photodetector with improved responsivity and response speed. Nanoscale.

[49] Ma N., Lu C., Liu Y., Han T., Dong W., Wu D., Xu X. (2024). Direct Z-Scheme Heterostructure of Vertically Oriented SnS_2_ Nanosheet on BiVO_4_ Nanoflower for Self-Powered Photodetectors and Water Splitting. Small.

[50] Li X., Li Y., Shen J., Ye M. (2016). A controlled anion exchange strategy to synthesize Bi_2_S_3_ nanoparticles/plate-like Bi_2_WO_6_ heterostructures with enhanced visible light photocatalytic activities for Rhodamine B. Ceram. Int..

[51] Taghinejad M., Xia C., Hrton M., Lee K.T., Kim A.S., Li Q., Guzelturk B., Kalousek R., Xu F., Cai W. (2023). Determining hot-carrier transport dynamics from terahertz emission. Science.

