# Supplementary material for: Enhanced Photoelectrochemical Performance of 2D Bi2O3/TiO2 Heterostructure Film by Bi2S3 Surface Modification and Broadband Photodetector Application
Source: Materials (Basel). 2025 Jul 28;18(15):3528. doi: 10.3390/ma18153528 (PMC12348803; doi:10.3390/ma18153528)
Supplement: Supplementary file 1 [file materials-18-03528-s001.zip › materials-3758880-supplementary.pdf]

Supporting Information

Enhanced photoelectrochemical performance of 2D Bi_2O_3/TiO_2 heterostructure film by Bi_2S_3 surface modification and broadband photodetector application

Lai Liu[a], Huizhen Yao[b]*

a Key Laboratory of Instrumentation Science and Dynamic Measurement, Ministry of Education, School of Instrument and Electronics, North University of China, Taiyuan 030051, China

b Key Laboratory of Micro/Nano Devices and Systems, School of Semiconductor and Physics, North University of China, Taiyuan, 030051, China

* Corresponding author: Huizhen Yao

E-mail address: huizhenyao@nuc.edu.cn

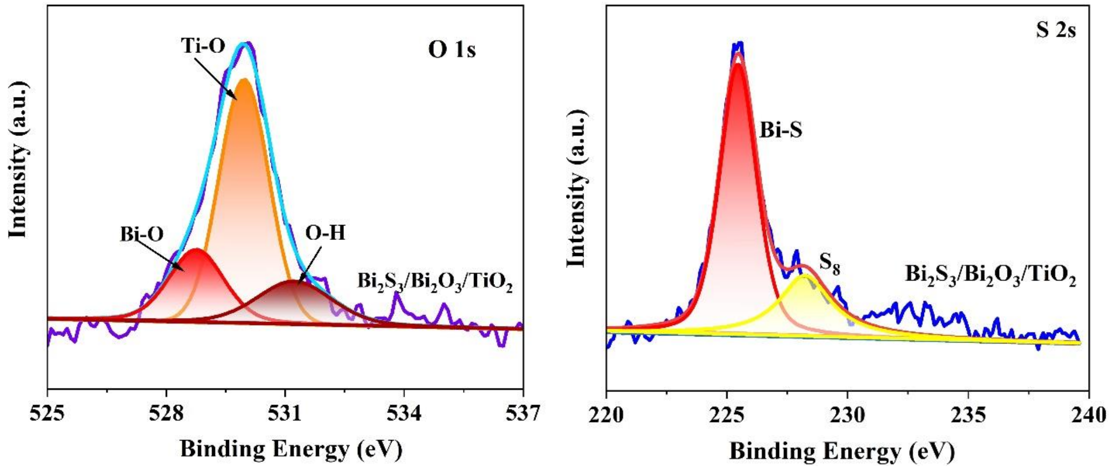

Fig. S1. High-resolution XPS spectra of O 1s and S 2s in $Bi_2S_3/Bi_2O_3/TiO_2$ film.

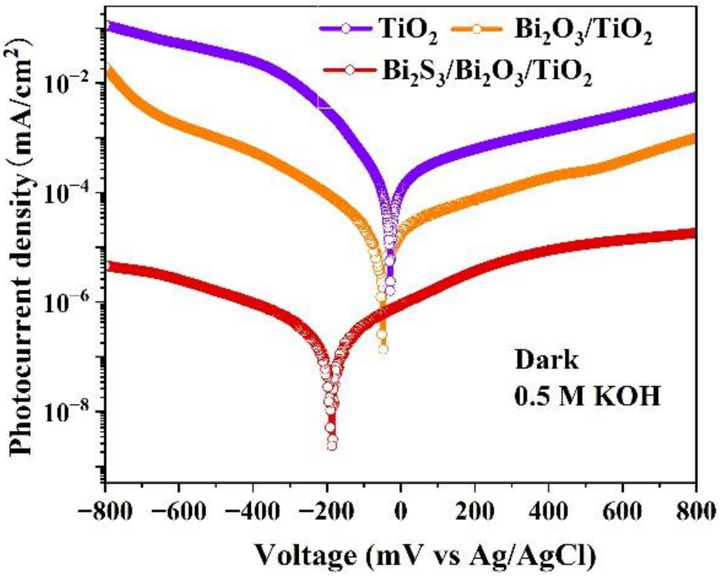

Fig. S2. LSV curves under dark.

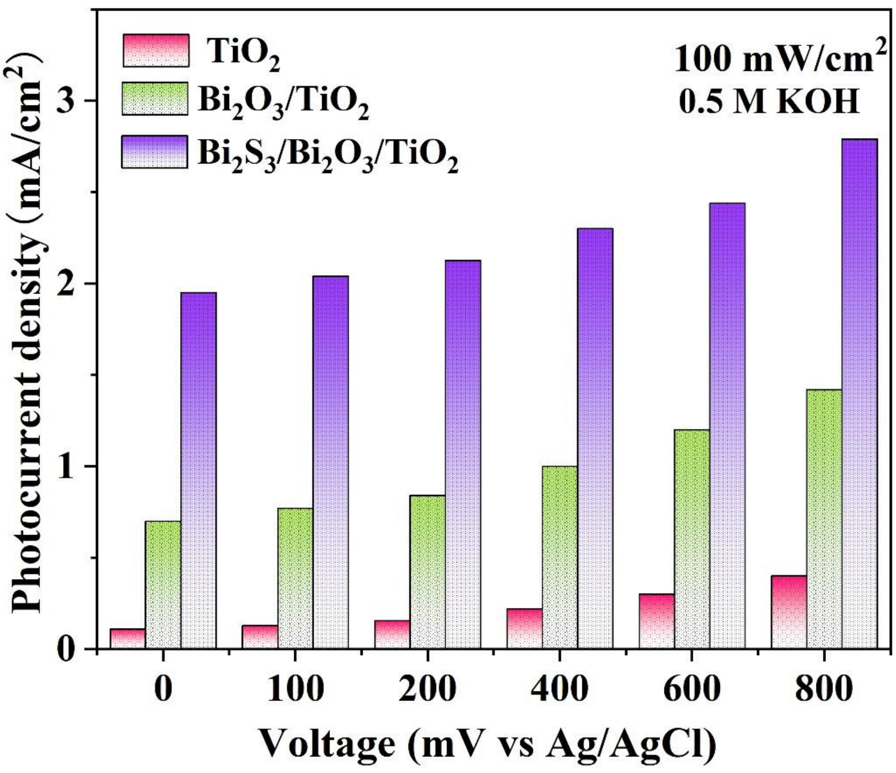

Fig. S3. Histogram of photocurrent density at various bias potential for different PEC devices.

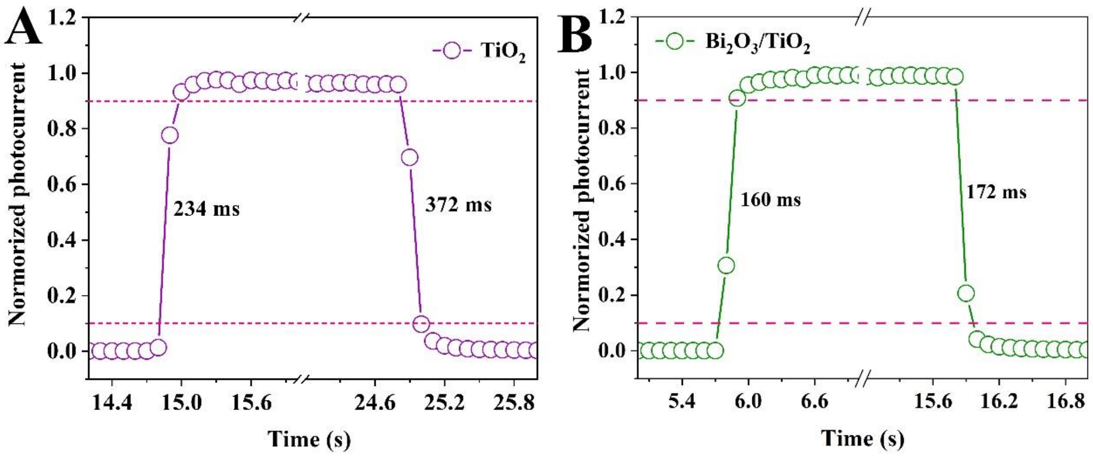

Fig. S4. Transient photocurrent at 0 V bias of (A) pristine TiO_2 and Bi_2O_3/TiO_2 film.

Table S1 Calculated E_{CB} and E_{VB} of TiO_2, Bi_2O_3 and Bi_2S_3

Semiconductors	E_g	X	E_{cB}	E_{vB}
TiO_2	3.25 eV	5.81	-0.32	2.94
Bi_2O_3	2.83	6.23	0.31	3.15
Bi_2S_3	1.58	5.27	-0.02	1.56

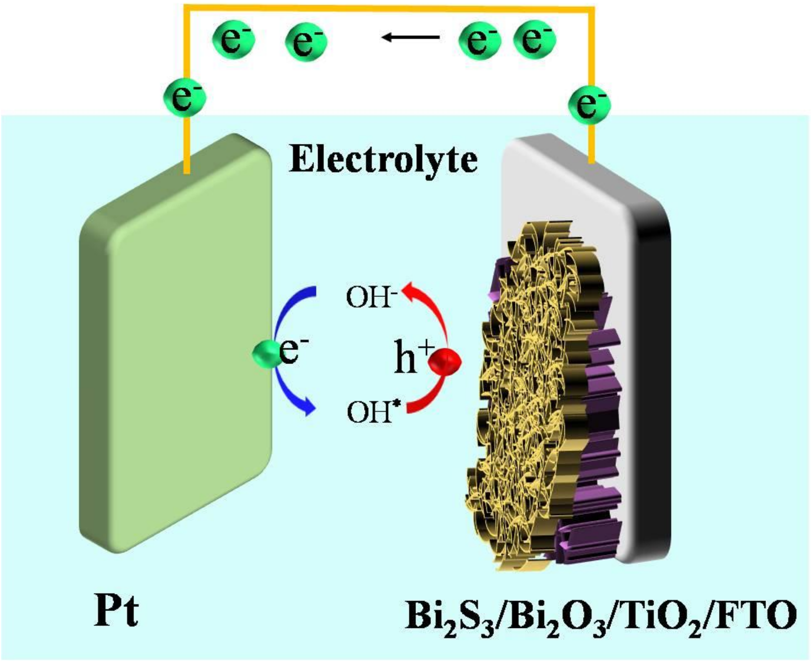

Fig. S5. Schematic illustration of PEC photodetector based on $Bi_2S_3/Bi_2O_3/TiO_2$ composites film.
